# Noninvasive prediction of lymph node metastasis in pancreatic cancer using an ultrasound-based clinicoradiomics machine learning model

**DOI:** 10.1186/s12938-024-01259-3

**Published:** 2024-06-18

**Authors:** Dong-yue Wen, Jia-min Chen, Zhi-ping Tang, Jin-shu Pang, Qiong Qin, Lu Zhang, Yun He, Hong Yang

**Affiliations:** 1 Department of Medical Ultrasonics, Guangxi Zhuang Autonomous Region, First Affiliated Hospital of Guangxi Medical University, Nanning, 530021 People’s Republic of China; 2 Department of Medical Pathology, Guangxi Zhuang Autonomous Region, First Affiliated Hospital of Guangxi Medical University, Nanning, 530021 People’s Republic of China

**Keywords:** Ultrasound, Radiomics, Machine learning, Pancreatic cancer, Lymph node metastasis

## Abstract

**Objectives:**

This study was designed to explore and validate the value of different machine learning models based on ultrasound image-omics features in the preoperative diagnosis of lymph node metastasis in pancreatic cancer (PC).

**Methods:**

This research involved 189 individuals diagnosed with PC confirmed by surgical pathology (training cohort: *n* = 151; test cohort: *n* = 38), including 50 cases of lymph node metastasis. Image-omics features were extracted from ultrasound images. After dimensionality reduction and screening, eight machine learning algorithms, including logistic regression (LR), support vector machine (SVM), K-nearest neighbors (KNN), random forest (RF), extra trees (ET), extreme gradient boosting (XGBoost), light gradient boosting machine (LightGBM), and multilayer perceptron (MLP), were used to establish image-omics models to predict lymph node metastasis in PC. The best omics prediction model was selected through ROC curve analysis. Machine learning models were used to analyze clinical features and determine variables to establish a clinical model. A combined model was constructed by combining ultrasound image-omics and clinical features. Decision curve analysis (DCA) and a nomogram were used to evaluate the clinical application value of the model.

**Results:**

A total of 1561 image-omics features were extracted from ultrasound images. 15 valuable image-omics features were determined by regularization, dimension reduction, and algorithm selection. In the image-omics model, the LR model showed higher prediction efficiency and robustness, with an area under the ROC curve (AUC) of 0.773 in the training set and an AUC of 0.850 in the test set. The clinical model constructed by the boundary of lesions in ultrasound images and the clinical feature CA199 (AUC = 0.875). The combined model had the best prediction performance, with an AUC of 0.872 in the training set and 0.918 in the test set. The combined model showed better clinical benefit according to DCA, and the nomogram score provided clinical prediction solutions.

**Conclusion:**

The combined model established with clinical features has good diagnostic ability and can be used to predict lymph node metastasis in patients with PC. It is expected to provide an effective noninvasive method for clinical decision-making, thereby improving the diagnosis and treatment of PC.

## Introduction

Pancreatic cancer (PC) is currently one of the most common highly lethal malignancies, with a 5-year survival rate of 12%. It is expected to become the third leading cause of cancer-related deaths by 2025 and is characterized by high malignancy, rapid progression, and extremely poor prognosis [1, 2]. Approximately 80% of PC cases are already in advanced or locally advanced stages when detected, and currently, most treatment options have limited effectiveness, leading to poor overall prognosis [3]. Lymph node metastasis is one of the vital factors affecting the prognosis of PC [4]. If the lymph node metastasis status of PC can be accurately determined before surgery, targeted treatment plans can be developed for patients' subsequent care. Therefore, accurate preoperative assessment of lymph node metastasis in PC patients is particularly important clinically. Currently, preoperative imaging of pancreatic tumors includes ultrasound, magnetic resonance imaging (MRI), and computed tomography (CT), all of which play an important role in PC diagnosis, treatment evaluation, prognosis prediction, etc. However, conventional imaging features can provide limited clinical information and cannot comprehensively and accurately reflect the tumor's characteristics. Radiomics can extract high-throughput imaging data from routine imaging images that are unrecognizable to the human eye, capture characteristic data reflecting tumor heterogeneity noninvasively, and integrate these features with clinical information for comprehensive analysis, thereby improving treatment efficacy and patient prognosis [5, 6]. Machine learning, as a new method based on computer data analysis, can learn from patterns in the dataset to discover more interactions between variables and outcomes and has been widely applied in clinical medical research [7–9]. Recent research has explored the utilization of radiomics and machine learning in pancreatic tumors, leveraging the advancements in artificial intelligence and machine learning algorithms [10–12]. These studies have shown that radiomics machine learning prediction models have stability, effectiveness, and high accuracy, demonstrating guiding significance for personalized and precise treatment of PC patients; the models are helpful in solving clinical problems and optimizing treatment plans. However, there is currently no research on different machine learning models based on ultrasound radiomics and clinical information parameters for the preoperative prediction of lymph node metastasis in PC. In this investigation, we selected different machine learning methods for radiomics models, developing and validating combined models based on ultrasound radiomics and clinical features for noninvasive preoperative prediction of lymph node metastasis status in PC patients.

## Results

### Extraction and selection of radiomics features

**Fig. 1:**
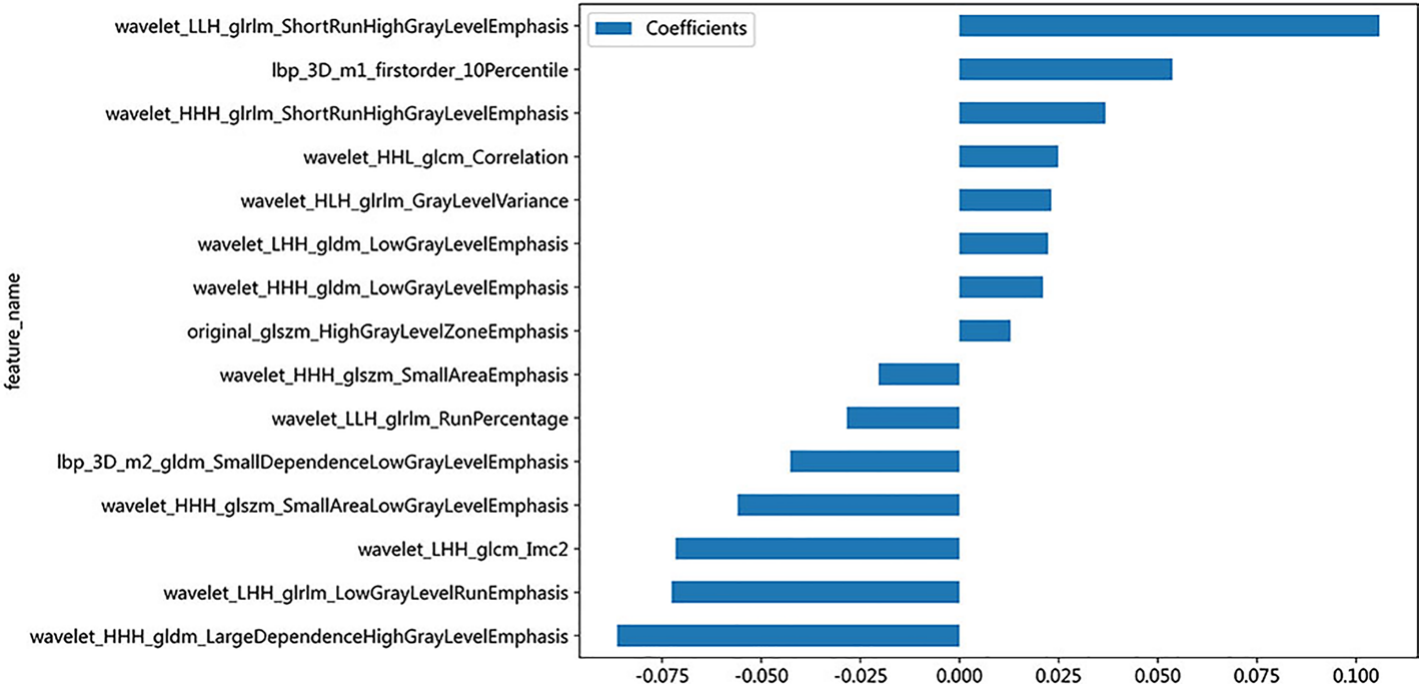
A histogram of radiographic scoring based on the final selected radiomics features

Based on the inclusion and exclusion criteria of this study, a total of 189 patients with PC confirmed by postoperative pathological findings were enrolled (102 males and 87 females), including 50 patients with lymph node metastasis (25 males and 25 females). These patients were randomly assigned to a training group and a testing group in an 8:2 ratio, with 151 cases in the training group and 38 cases in the testing group. We extracted 1561 high-throughput radiomics features from the region of interest (ROI) of each patient's ultrasound images and normalized the quantized radiomics features with Z score regularization. Through the Pearson correlation test and principal component analysis (PCA) of feature data, we used the least absolute shrinkage and selection operator (LASSO) regression and mean squared error (MSE) algorithm to finally determine 15 nonzero coefficient radiomics features (Fig. 1). Rad score is shown as follows:

$$ {\text{Rad score }} = {\text{ }}0.{\text{2771177243528842 }} + 0.0{\text{53686 }}*{\text{ lbp}}\_{\text{3D}}\_{\text{m1}}\_{\text{firstorder}}\_{\text{1}}0{\text{Percentile}} - 0.0{\text{42696 }}*{\text{ lbp}}\_{\text{3D}}\_{\text{m}}^{{\text{2}}} \_{\text{gldm}}\_{\text{SmallDependenceLowGrayLevelEmphasis }} + 0.0{\text{12872 }}*{\text{ original}}\_{\text{glszm}}\_{\text{HighGrayLevelZoneEmphasis }} - 0.0{\text{86265 }}*{\text{ wavelet}}\_{\text{HHH}}\_{\text{gldm}}\_{\text{LargeDependenceHighGrayLevelEmphasis }} + 0.0{\text{21}}0{\text{36 }}*{\text{ wavelet}}\_{\text{HHH}}\_{\text{gldm}}\_{\text{LowGrayLevelEmphasis }} + 0.0{\text{36746 }}*{\text{ wavelet}}\_{\text{HHH}}\_{\text{glrlm}}\_{\text{ShortRunHighGrayLevelEmphasis }} - 0.0{\text{2}}0{\text{433 }}*{\text{ wavelet}}\_{\text{HHH}}\_{\text{glszm}}\_{\text{SmallAreaEmphasis }} - 0.0{\text{55978 }}*{\text{ wavelet}}\_{\text{HHH}}\_{\text{glszm}}\_{\text{SmallAreaLowGrayLevelEmphasis }} + 0.0{\text{249}}0{\text{3 }}*{\text{ wavelet}}\_{\text{HHL}}\_{\text{glcm}}\_{\text{Correlation }} + 0.0{\text{23112 }}*{\text{ wavelet}}\_{\text{HLH}}\_{\text{glrlm}}\_{\text{GrayLevelVariance }} - 0.0{\text{71616 }}*{\text{ wavelet}}\_{\text{LHH}}\_{\text{glcm}}\_{\text{Imc2 }} + 0.0{\text{22336 }}*{\text{ wavelet}}\_{\text{LHH}}\_{\text{gldm}}\_{\text{LowGrayLevelEmphasis }} - 0.0{\text{72566 }}*{\text{ wavelet}}\_{\text{LHH}}\_{\text{glrlm}}\_{\text{LowGrayLevelRunEmphasis }} - 0.0{\text{284}}00{\text{ }}*{\text{ wavelet}}\_{\text{LLH}}\_{\text{glrlm}}\_{\text{RunPercentage }} + 0.{\text{1}}0{\text{5875 }}*{\text{ wavelet}}\_{\text{LLH}}\_{\text{glrlm}}\_{\text{ShortRunHighGrayLevelEmphasis}} $$

### Construction of the radiomics model

**Fig. 2:**
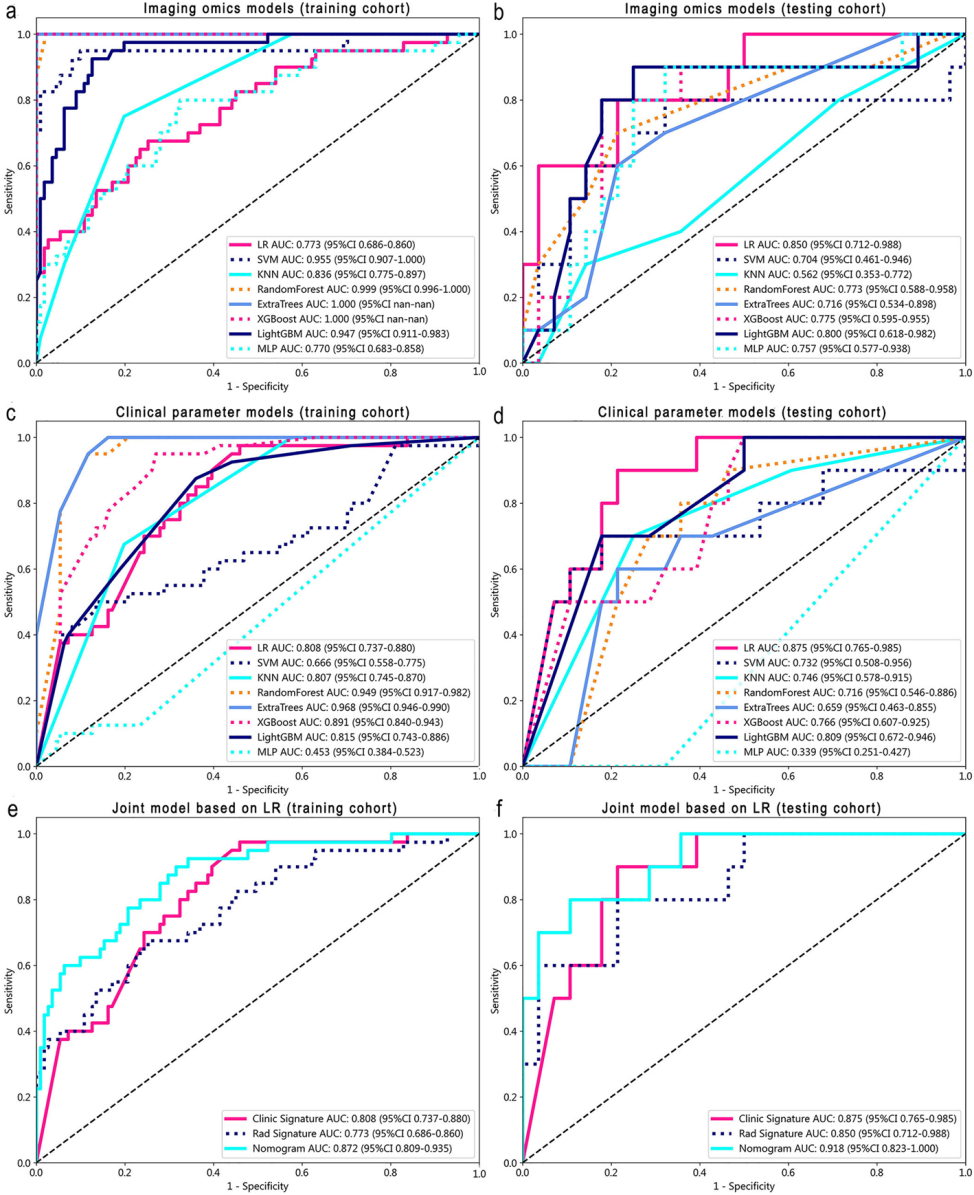
Receiver operating characteristic (ROC) curve set of radiomics-clinical parameter features. ROC curve set of radiomics models on the training **a** and testing **b** sets. ROC curve set of clinical parameter models on the training **c** and testing **d** cohorts. E. Collection of ROC curves of training **e** and testing **f** sets based on the logistic regression (LR) machine learning algorithm joint model

Eight machine learning algorithms (including logistic regression (LR), support vector machine (SVM), K-nearest neighbors (KNN), random forest (RF), extra trees (ET), extreme gradient boosting (XGBoost), light gradient boosting machine (LightGBM), and multilayer perceptron (MLP) were used to construct radiomics models on the training group and validate them on the testing group. Among the constructed radiomics models, the logistic regression model showed better predictive performance in the testing dataset (AUC = 0.850, 95% CI 0.712–0.989). The ET and XGBoost models in both the training and testing groups showed overfitting trends. To ensure the effectiveness and stability of the model, the LR model was ultimately selected as the best radiomics model (Fig. 2a, 2b).

### Construction of the clinical model

In the training group, significant differences were observed in the location distribution, echogenicity, boundary, and tumor markers CEA and CA199 between PC patients with or without lymph node metastasis in ultrasound images. In the testing group, statistically significant differences were found in the boundary of lesions, serum amylase, and CA199 in the images (Table 1). Univariate and multivariate logistic regression analyses showed that the boundary of lesions in 2D ultrasound images (OR = 1.276, 95% CI 1.142–1.426) and the tumor marker CA199 (OR = 1.042, 95% CI 1.116–1.318) were independent predictors of lymph node metastasis in PC (Table 2). Using these two clinical feature parameters, a clinical machine learning model was constructed, and the logistic regression model showed better predictive performance in the testing group (AUC = 0.875, 95% CI 0.765–0.986), outperforming the other seven machine learning models (Fig. 2c, 2d).

**Table 1:** Characteristics of pancreatic cancer patients in the training and testing datasets regarding lymph node metastasis

Variable		Total (*N* = 189)	Training dataset (*N* = 151)	Testing dataset (*N* = 38)	*P* value
Age (years)		58.00 ± 11.35	57.32 ± 11.68	60.68 ± 9.58	0.144
CEA (ng/ml)		40.43 ± 304.30	45.44 ± 339.34	20.52 ± 56.14	0.803
CA125 (U/ml)		109.47 ± 242.72	96.81 ± 220.78	159.76 ± 313.83	0.735
CA153 (U/ml)		23.82 ± 33.77	24.00 ± 36.97	23.07 ± 15.96	0.276
CA199 (U/ml)		2661.07 ± 4409.41	2380.49 ± 4215.79	3775.97 ± 5014.07	0.536
AFP (ng/ml)		3.49 ± 4.65	3.56 ± 5.14	3.21 ± 1.61	0.887
Serum amylase (U/l)		266.84 ± 539.09	286.58 ± 597.47	188.38 ± 146.96	0.953
Duration (days)		64.71 ± 78.08	60.63 ± 73.12	80.94 ± 94.66	0.097
Maximum diameter (cm)		3.80 ± 1.48	3.82 ± 1.49	3.72 ± 1.48	0.885
Gender (*n*, %)	Female	87 (46.03)	71 (47.02)	16 (42.11)	0.718
	Male	102 (53.97)	80 (52.98)	22 (57.89)	
Location (*n*, %)	Head/ neck	137 (72.49)	107 (70.86)	30 (78.95)	0.427
	Body/ tail	52 (27.51)	44 (29.14)	8 (21.05)	
Echo (*n*, %)	High/mixed	41 (21.69)	33 (21.85)	8 (21.05)	1.000
	Hypoecho	148 (78.31)	118 (78.15)	30 (78.95)	
Boundary (*n*, %)	Clear	79 (41.80)	65 (43.05)	14 (36.84)	0.611
	Unclear	110 (58.20)	86 (56.95)	24 (63.16)	
Differentiation (*n*, %)	High/ Moderate	164 (86.77)	133 (88.08)	31 (81.58)	0.430
	Low	25 (13.23)	18 (11.92)	7 (18.42)	

Values are presented as number (%), mean (SD) or median (IQR)

*CEA* carcino-embryonic antigen, *CA* cancer antigen, *AFP* alpha fetoprotein

**Table 2:** Univariate and multivariate logistic regression analysis used for selecting clinical machine learning model development

Variable	Univariate analysis		Multivariate analysis	
	OR (95% *CI*)	*p* value	OR (95% *CI*)	*p* value
Gender	0.959 (0.862–1.067)	0.514		
Age (years)	1.005 (1.002–1.010)	0.079		
CEA (ng/ml)	1.001 (1.002–1.009)	0.033	1.000 (1.003–1.006)	0.108
CA125 (U/ml)	1.064 (1.001–1.003)	< 0.001	1.000 (1.004–1.005)	0.223
CA153 (U/ml)	1.001 (0.999–1.002)	0.554		
CA199 (U/ml)	1.151 (1.001–1.010)	< 0.001	1.042 (1.116–1.318)	< 0.001
AFP (ng/ml)	0.993 (0.982–1.004)	0.313		
Serum amylase (U/l)	1.000 (1.001–1.008)	0.649		
Duration (days)	1.000 (0.999–1.000)	0.213		
Maximum diameter (cm)	1.001 (0.966–1.038)	0.949		
Location	0.858 (0.763–0.966)	0.034	0.911 (0.823–1.008)	0.131
Echo	1.317 (1.163–1.493)	< 0.001	1.051 (0.931–1.188)	0.499
Boundary	1.476 (1.339–1.627)	< 0.001	1.276 (1.142–1.426)	< 0.001
Differentiation	1.224 (1.048–1.430)	0.033	1.024 (0.889–1.179)	0.782

*CEA* carcino-embryonic antigen, *CA* cancer antigen, *AFP* alpha fetoprotein

### Construction and evaluation of the combination model

**Fig. 3:**
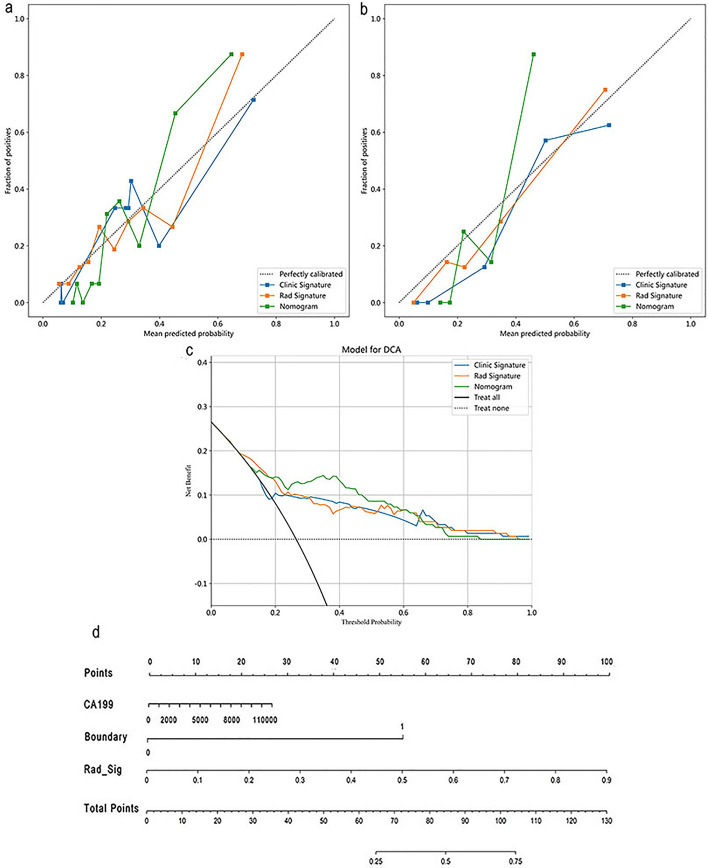
Calibration curves in the training **a** and testing **b** sets. Decision curve analysis (DCA) of radiomics, clinical parameters, and joint models in the testing **c** cohort. Clinical application of omics model in lymph node metastasis of pancreatic cancer **d**

By integrating the 15 radiomics features and clinically independent predictors (boundary of lesions and tumor marker CA199), we developed a combination model using the LR machine learning model that was used to predict the lymph node metastasis status of PC patients before surgery. In the training and testing groups, the combination model outperformed the radiomics and clinical machine learning models, demonstrating the best predictive performance (training group AUC = 0.872, 95% CI 0.809–0.935; testing group AUC = 0.918, 95% CI 0.823–1.000) (Fig. 2e, 2f). The Hosmer‒Lemeshow test indicated good consistency of the calibration curves of the combination model in the training and testing datasets, with radiomics features (*P* = 0.761), clinical information features (*P* = 0.801), and the combined model (*P* = 0.160) (Fig. 3a, 3b). We also evaluated each model using decision curve analysis (DCA), and the combination model showed better clinical utility in the preoperative prediction of lymph node metastasis in PC (Fig. 3c). The clinical application nomogram score sheet displayed the clinical predictive scheme (Fig. 3d).

## Discussion

PC is characterized by its insidious onset, atypical early symptoms and signs, high malignancy, rapid progression, and poor prognosis [13, 14]. The sole effective approach for PC patients to acquire an opportunity for healing and prolonged survival is surgical resection. Radical surgery includes resection of the primary tumor and regional lymph node clearance, with lymph node metastasis being an important factor affecting surgical prognosis [15]. Since lymph node metastasis (N stage) is determined by surgical pathology, it is clinically significant to be able to safely and accurately predict lymph node metastasis preoperatively through the analysis and modeling of PC ultrasound images and clinical features. In this research, a total of eight distinct machine learning techniques were employed (namely LR, SVM, KNN, RF, ET, XGBoost, LightGBM, and MLP) to construct and verify a merged model utilizing ultrasound radiomics and clinical characteristics, aiming to predict lymph node metastasis status in PC patients with high diagnostic performance, reveal the N stage of PC, and provide effective information for individualized and precise treatment of PC patients.

At present, there is no consensus on the prediction of lymph node metastasis in PC based on imaging and clinical information features. Therefore, a new approach or method is needed to assist clinical decision-making. Ultrasound, due to its convenience, noninvasiveness, lack of radiation, and ability to observe multiple axes, is a frontline diagnostic tool in clinical practice and is generally used for the initial diagnosis and follow-up of PC [16]. However, the clinical information provided solely by traditional ultrasound image features is limited, making it difficult to differentiate lymph node metastasis and fully and accurately reflect the characteristics of pancreatic tumors. Radiomics is a noninvasive method that mainly reflects the heterogeneity of tumors by extracting high-throughput features from images. It can be used alone or in combination with histology, genomics, and proteomics data to solve clinical problems [17–19]. The application of a combined model based on radiomics and clinical parameters provides a new approach and insights for establishing prediction models with multiple features. Zheng and colleagues discovered that a fusion of radiomics and clinical characteristics exhibited good diagnostic capability in distinguishing between benign and malignant tumors in the parotid gland. This approach is anticipated to offer a noninvasive and efficient means for clinical decision-making, as stated in reference [20]. Li et al. also reported that a combined feature model can noninvasively distinguish autoimmune pancreatitis from PC [10]. The findings of this study suggest that the combination of the radiomics texture feature model and clinical information feature machine learning model has higher diagnostic performance than models constructed with individual features alone. The combined model can not only predict the lymph node metastasis status of PC patients comprehensively but also improve the performance and accuracy of prediction, which is consistent with the results of Pszczolkowski's investigation [21].

In the field of medicine, machine learning has shown great potential with its excellent computing capabilities in image reconstruction, segmentation, recognition, and classification. Using high-throughput imaging techniques to obtain image information and using machine learning as the main computational tool, the ‘‘radiomics + machine learning’’ analytical approach has become the mainstream solution for medical image analysis at this stage. Compared with traditional analytical methods, the ‘‘radiomics + machine learning’’ mode has the ability to learn from historical image data, reducing the interference of subjective factors and ensuring the objectivity and reliability of the prediction results [22]. Van et al. used spline interpolation sampling, Z score normalization, and PCA dimension reduction to clean and process feature data and used a multilayer perceptron classifier algorithm to achieve automated diagnosis of Ménière's disease [23]. Zaragori et al. processed feature data using hierarchical clustering, Spearman correlation analysis, and rank-sum test methods and used LR, neural network, RF, and SVM classifiers to predict IDH mutations and lp/19q codeletions in brain gliomas [24]. The above studies used various methods for feature selection and classification. By constructing multiple machine learning algorithm models and comparing their diagnostic performance, the accuracy and sustainability of the models can be better demonstrated. In this study, the LR model had the best comprehensive prediction performance (training set AUC = 0.872, test set AUC = 0.918). This may be attributed to the fact that the LR model is designed to binarize data by maximizing the likelihood function and using gradient descent. Additionally, this model is more sensitive to multicollinear data and imbalanced data [25, 26].

In addition to high-throughput ultrasound imaging data, the dataset of this study also included participants' clinical information (clinical examination results, laboratory test information, pathological differentiation) as covariates in the analysis of radiomics machine models to improve the accuracy and precision of research predictions. This study is a retrospective single-center study with multiple factors affecting the research results, such as sample size, parameter acquisition, and extraction methods. Hence, it is crucial to have larger sample sizes, prospective studies, and involve multiple centers to authenticate the outcomes of the model and enhance its effectiveness.

In summary, our research and development have validated a machine learning model based on ultrasound that integrates and extracts high-dimensional data information from ultrasound images. This model has excellent diagnostic capabilities and can be used to predict the lymph node metastasis status in PC patients. It is expected to provide a noninvasive and effective approach for clinical decision-making, thus improving the level of diagnosis and treatment of PC.

### Materials and methods

**Fig. 4:**
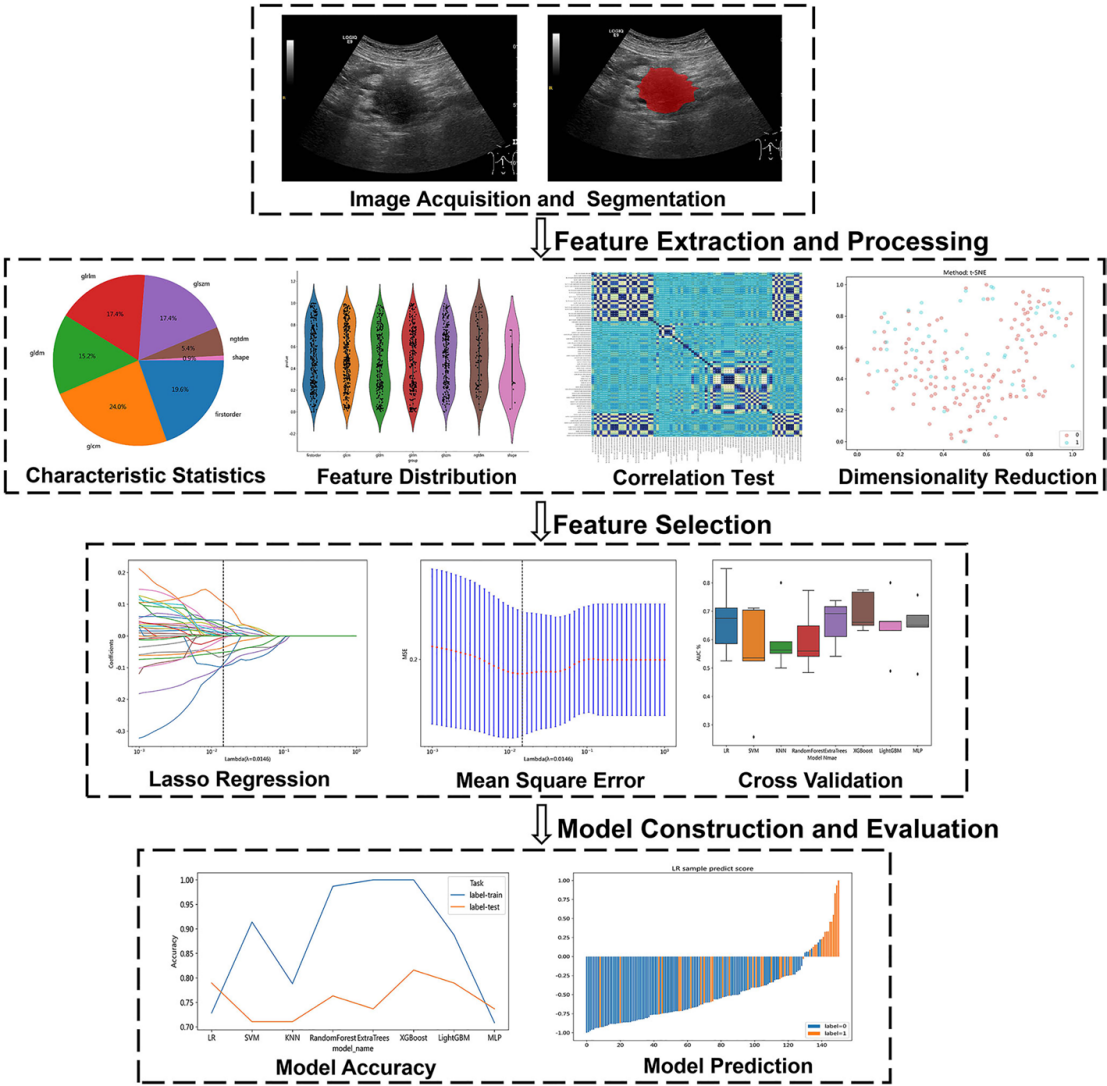
The machine learning workflow diagram based on ultrasound-based imaging omics

The main research steps of this study include acquiring and preprocessing two-dimensional ultrasound images of PC, calibrating the tumor region of interest, extracting and reducing the dimensionality of radiomics features, analyzing clinical parameter data, and establishing and evaluating prediction models (Fig. 4). We first explored the performance of the machine model based on ultrasound radiomics features in predicting lymph node metastasis of PC. To further explore the potential biological mechanism of PC N staging, we also attempted to evaluate clinical tumor marker features: CEA, CA125, CA153, and CA199. By constructing a combined model, we aimed to achieve noninvasive prediction of lymph node metastasis in PC.

### Patient population

**Fig. 5:**
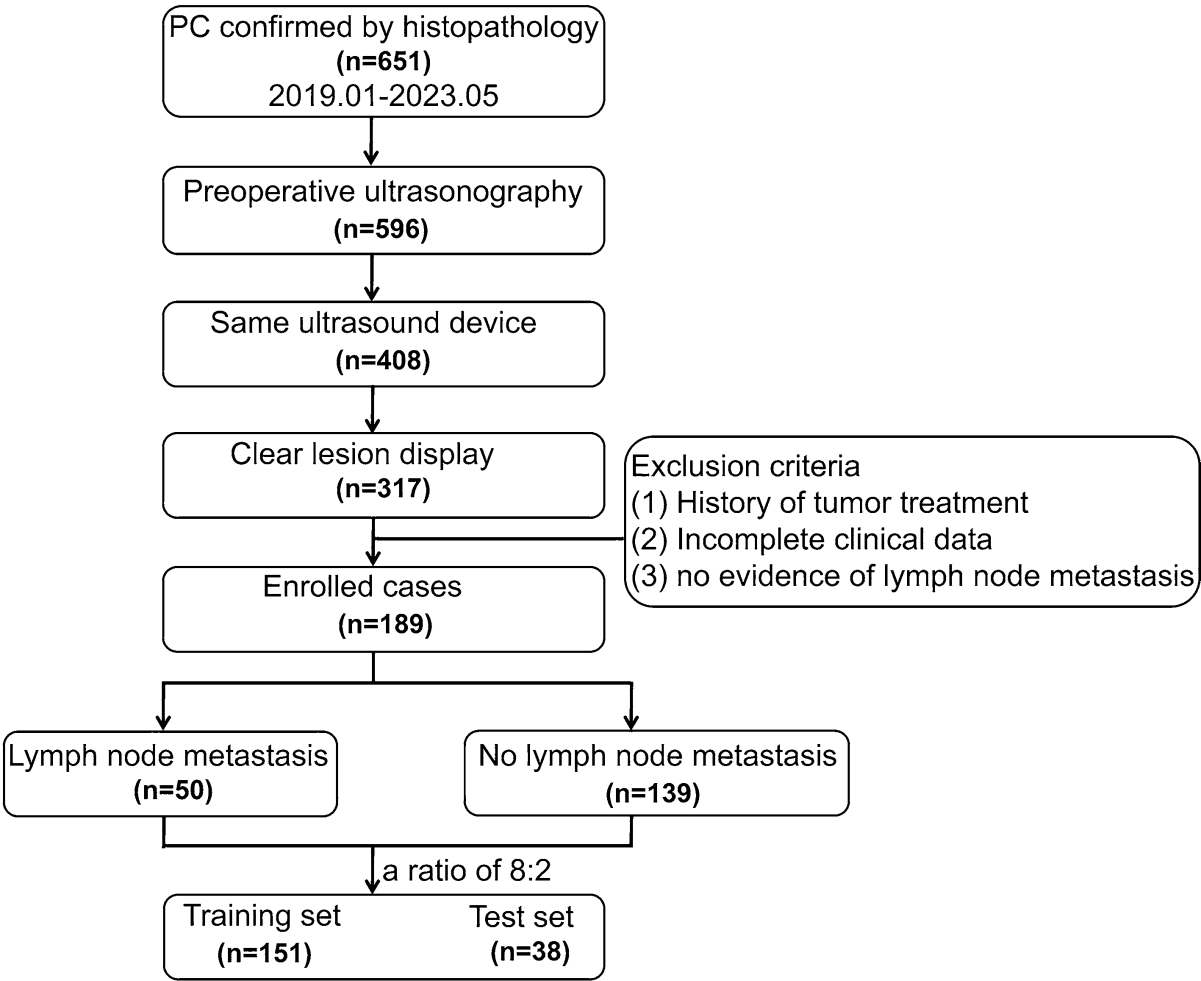
The workflow diagram of inclusion and exclusion criteria for this study

This research was approved by the Ethics Committee. The medical data of patients with PC who underwent surgical treatment at this medical institution from January 2019 to May 2023 were retrospectively analyzed. The inclusion criteria were as follows: (1) Histopathologically confirmed PC; (2) preoperative comprehensive ultrasound examination; (3) same ultrasound equipment used with the same parameters for scanning; (4) clearly and unequivocally displayed target lesion on ultrasound images. The exclusion criteria were as follows: (1) history of tumor treatment before surgery; (2) incomplete clinical data; and (3) lack of detailed lymph node metastasis staging information in pathological reports (Fig. 5).

### Image acquisition

We used the GE Logiq E9 color ultrasound diagnostic instrument (C5-1 convex array probe, 2.8–5.0 MHz) to acquire images, maintaining the room temperature within the range of 22–28 degree. The probe was sufficiently covered with a coupling agent to eliminate gas interference during scanning. Patients were needed to fast for 8–12 h before the examination and were placed in the supine or lateral position. The sequential parallel section method was used to observe and analyze the overall conditions of PC lesions, clearly displaying the maximum cross-sectional area of the lesions, and the images were saved in Digital Imaging and Communications in Medicine (DICOM) format.

### Lesion segmentation

Two ultrasound doctors (with 8 and 21 years of diagnostic experience) independently used ITK-SNAP software (http://www.itksnap.org) to trace the contours of PC lesions along the edge of the maximum cross-sectional area of each lesion, determining the ROI of the tumor. To ensure the stability and repeatability of the extracted radiomics features, the intraclass correlation coefficient (ICC) was employed to evaluate the intra- and interobserver consistency of features between senior and junior doctors. A value of ICC above 0.9 suggests strong consistency.

### Ultrasound image and clinical data analysis

Patient ultrasound images and clinical data were obtained from the Picture Archiving and Communication System (PACS) of our hospital. We retrospectively analyzed the distribution, maximum diameter, echogenicity, and border of ultrasound images as well as clinical parameters, including sex, age, course of disease, serum amylase, tumor markers (CEA, CA125, CA153, CA199, AFP), and postoperative pathological diagnosis.

### Image feature selection and extraction

Radiomics features provide objective high-throughput imaging features for quantitative analysis of intralesional heterogeneity and biological characteristics. The ultrasound images were processed for quantitative feature extraction using Ultimate software (GE healthcare). The extracted radiomics features included first-order features, gray-level cooccurrence matrix (GLCM), gray-level dependence matrix (GLDM), gray-level run-length matrix (GLRLM), gray-level size zone matrix (GLSZM), neighborhood gray-tone difference matrix (NGTDM), and shape features. Features with zero variance were removed, and Z score standardization was applied. Redundant features with a Pearson correlation coefficient (corr) greater than 0.9 were eliminated. PCA was used for feature dimensionality reduction. The LASSO regression model with tenfold cross-validation and MSE as penalty parameters were used to determine the optimal λ value as the regularization coefficient, and radiomics features with nonzero coefficients were selected.

### Construction and validation of the radiomics models

Radiomics models were constructed on the training set using various classifiers, including LR, SVM, KNN, RF, ET, XGBoost, LightGBM, and MLP [26–33]. To evaluate the accuracy of the radiomics features in predicting PC lesions, the generated radiomics models were evaluated using the testing set, and the diagnostic performance of the models was quantitatively compared using AUC.

### Construction and clinical application evaluation of clinical and combined models

Univariate logistic regression analysis was performed on the features of ultrasound images and clinical pathological parameters in the training dataset, and multivariate logistic regression was performed on significant indicators to obtain the predictive variables for the clinical model. A combined model was constructed by combining the best radiomics model and clinical features model. The performance of the constructed model was evaluated and validated using the test set. The clinical application value of the model was evaluated using the net benefit and DCA threshold probability nomogram.

### Statistical analysis

Statistical analysis was conducted using SPSS 25.0 (http://www.spss.com.hk/) and Python (https://www.python.org/) software. Continuous variables were described using a *t* test or Mann‒Whitney *U* test depending on whether they followed a normal distribution, with mean and standard deviation or median. Categorical data were described using the Chi-square test or Fisher’s exact test with rates. Multivariate analysis was performed using logistic regression models, and odds ratios (ORs) were used for description. All statistical tests were two tailed, and *p* < 0.05 was considered statistically significant.

## Data Availability

All the data supporting our findings can be found in the Results section of the paper. Please contact authors for data request.

## Abbreviations

PCA: Principal component analysis
LASSO: Least absolute shrinkage and selection operator
MSE: Mean square error
SVM: Support vector machine
KNN: K-nearest neighbors
RF: Random forest
ET: Extra trees
XGBoost: Extreme gradient boosting
LightGBM: Light gradient boosting machine
MLP: Multilayer perceptron
DCA: Decision curve analysis
PC: Pancreatic cancer
ROI: Region of interest
ICC: Intraclass correlation coefficient
